# Sixteen-Frame Gated Myocardial Perfusion SPECT as a Surrogate for Equilibrium Radionuclide Angiography in Measurement of Systolic and Diastolic Indices: A Cross-Sectional Study

**DOI:** 10.1055/s-0044-1788334

**Published:** 2024-07-11

**Authors:** Toktam Hosseinnezhad Ariani, Mohammadali Ghodsirad, Faezeh Hosseinnejad Ariani, Hoorak Poorzand, Ramin Sadeghi, Vahid Reza Dabbagh Kakhki

**Affiliations:** 1 Nuclear Medicine Research Center, Mashhad University of Medical Sciences, Mashhad, Iran; 2 Department of Nuclear Medicine, Shohada Tajrish Hospital, School of Medicine, Shahid Beheshti University of Medical Sciences, Tehran, Iran; 3 Metabolomics and Genomics Research Center, Endocrinology and Metabolism Molecular-Cellular Sciences Institute, Tehran University of Medical Sciences, Tehran, Iran; 4 Department of Cardiovascular, Imam Reza Hospital, Mashhad University of Medical Sciences, Mashhad, Iran; 5 Department of Nuclear Medicine, Ghaem Hospital, Mashhad University of Medical Sciences, Mashhad, Iran

**Keywords:** equilibrium radionuclide angiography, myocardial perfusion SPECT, ventricular diastolic function

## Abstract

**Introduction** Equilibrium radionuclide angiography (ERNA) has long been assumed as the preferred method to assess cardiac volumes as well as left ventricular systolic and diastolic indices. ERNA was used to diagnose subtle changes in cardiac function during chemotherapy or early stages of heart failure. Gated myocardial perfusion SPECT (GMPS) was introduced as a more feasible and versatile alternative to ERNA, but the precision of GMPS to assess systolic and diastolic indices has not yet been fully reviewed.

**Method** We studied the left ventricular systolic and diastolic functional indices measured by a 16-frame GMPS and compared the results with those of ERNA in 25 patients. All the images were analyzed visually, semi-quantitatively, and quantitatively using quantitative gated SPECT (QGS), quantitative blood pool SPECT (QBS), and planar gated blood pool (PGBP) software. The left ventricular functional indices calculated using QGS compared with those obtained using QBS and PGBP

**Result** Our study found a significant correlation between the left ventricular ejection fraction (LVEF) calculated using the PGBP, QGS, and QBS methods. There was a significant correlation between the LV peak ejection rate (LVPER) calculated by the PGBP and QGS analyses, and there was no significant difference in the LVPER calculated with the QGS and QBS methods. This study also revealed a significant correlation between the LV peak filling rate (LVPFR) calculated by QBS and QGS, with no significant difference between them. We also found a significant correlation between LV end systolic volume (LVESV) calculated using QGS and QBS and between LV end diastolic volume (LVEDV) calculated using QGS and QBS software. This study also revealed a significant correlation between the LV mean filling rate over the first third of diastole (LVMFR/3) calculated using the QGS and QBS software.

**Conclusion** Considering the significant correlation between LVEF, LVPER, LVPFR, LVESV, LVMFR/3, and LVEDV calculated using the QGS and QBS methods in our study, the 16-frame GMPS could be regarded as an acceptable substitute for ERNA in the investigation of systolic and diastolic indices.

## Introduction

Numerous studies have shown that left ventricular (LV) systolic and diastolic function, especially in coronary artery disease (CAD), can be a predictor of morbidity and mortality. 1 2 Diastolic dysfunction is more persistent than systolic dysfunction after acute ischemic heart disease. 3 4 A high percentage of patients with CAD, when evaluated by equilibrium radionuclide angiography (ERNA), show abnormal LV diastolic filling at rest, while LV systolic function is not affected, 5 which can be used as an early indicator of CAD.

In gated myocardial perfusion SPECT (GMPS), both myocardial perfusion and function, especially LV indices such as LV volumes and ejection fraction, are examined at the same time. 6 In addition, using existing software, LV diastolic function indices are also calculated. Evaluation of the LV function is a strong predictor of long-term outcomes after myocardial infarction (MI). 7 8

ERNA is an established “gold standard” or reference standard modality for the assessment of ventricular systolic and diastolic functions in nuclear medicine, as has been reported by numerous authors. 9 10 11 Information obtained from GMPS also has a high clinical value. 12 Various studies that compared the 8- and 16-frame gated SPECT in determining the systolic and diastolic parameters of the LV resulting from GMPS showed a higher precision of the 16-frame gated images. 9 12 13 In one study, the systolic indices of 8-, 16-, and 32-frame images were compared with the results of ERNA, which showed that the LV ejection fraction (LVEF) obtained from GMPS was underestimated compared with ERNA. This underestimation diminished by adding the number of gating frames. 9 14

Numerous studies have compared the functional indices of GMPS with the findings of nonstandard gold procedures, such as ultrasound echocardiography, and their results have been very varied and heterogeneous. 15 16

In this study, we evaluated the correlation between the systolic and diastolic indices obtained from GMPS and ERNA. Only a few studies have compared the data from ERNA obtained by quantitative blood pool SPECT (QBS) with GMPS obtained by quantitative gated SPECT (QGS), and most studies have ignored these valuable data. So diastolic indices obtained from GMPS are either ignored or omitted in the final report. Therefore, we aimed to validate the functional indices obtained from QGS to make use of them in patient care. 9 10 11 17

## Methods

After obtaining ethical approval from the Medical University Ethics Committee, 25 patients were studied. Written informed consent was obtained from all the participants.

### Patient Selection

Patients referred to the nuclear medicine department for GMPS were included in this study. Patients with any type of arrhythmia that prevented gated imaging and those who refused to undergo ERNA were excluded. After the 16-frame GMPS, the patients underwent planar and SPECT ERNA imaging.

### Technique and Acquisition

***GMPS*:** Forty-five minutes after intravenous injection of 20 to 25 mCi of ^99m^ Tc-sestamibi at rest, imaging was performed. Electrocardiogram (ECG) gated SPECT in the supine position was performed using a gamma camera equipped with low-energy high-resolution collimators. The gamma camera was a Siemens E.cam with two variable-angle heads and E. Soft software. The angle between the two collimators was set at 90 degrees and acquisition was performed in a 180-degree orbit, 32 frames from the right anterior oblique to the left posterior oblique positions. A 15% window cantered at 140-KeV photopeak was chosen. Gating was done using 16 frames per cardiac cycle and an acceptance window of 20%. The data obtained were stored in a 64 × 64 matrix. The images were reconstructed with filtered back projection and Butterworth filter (order: 5 and cutoff frequency: 0.55 cycles/pixel). Finally, after data processing with the QGS software, LV systolic and diastolic indices were recorded.

***ERNA*** : Within 3 to 10 days of the gated ^99m^ Tc-sestamibi SPECT study, patients underwent ERNA. Approximately 10 minutes after injection of 20-mCi ^99m^ Tc-labeled red blood cell (RBC; in vitro labeling method), 18 gated planar and SPECT blood pool images were obtained at rest. Gated planar blood pool images were acquired in the left anterior oblique using a large field of view equipped with a high-resolution parallel-hole, low-energy collimator. A zoom factor = 2 was used to acquire images in 64 × 64 matrices with 16 frames per cycle. A 30% energy window cantered at 140 keV was applied.

The gated SPECT blood pool study acquisition parameters were 16 consecutive frames with a 20% acceptance window, a 64 × 64 matrix, and a 15% window cantered on a 140-keV photopeak with a low-energy, high-resolution, parallel-hole collimator. The datasets were prefiltered using a Butterworth filter. These images were analyzed using QBS software. This software automatically fits the LV and right ventricular regions of interests (ROIs) and calculates the systolic and diastolic ventricular indices.

### Statistical Analysis

Patient demographics and GMPS and ERNA results are presented as mean ± standard deviation. Quantitative data were analyzed using the paired *t* -test, and qualitative data were analyzed using the chi-squared test. Analysis of variance (ANOVA) and repeated measures ANOVA were used to compare variables that were measured using different methods. All statistical analyses were performed using SPSS software. Spearman's correlation was used. A *p* -value less than 0.05 was considered significant.

Patients were evaluated for LV function using three methods, while some indices could be assessed using only two methods. Therefore, a paired *t* -test was used to compare these indicators. For LVEF, peak filling rate (PFR), and peak ejection rate (PER), which were measured using the three methods, the variance analysis was used.

## Results

Twenty-five patients, 18 men (72%) and 7 women (28%) with an age range of 36 to 73 years, were studied. Ten patients (40%) had a history of hypertension, 6 patients (24%) had diabetes mellitus, 10 patients (40%) had hyperlipidemia, 4 patients (16%) were smokers, 23 patients (92%) were symptomatic, and 5 patients (20%) had no chest pain. Among the symptomatic patients, 11 patients (44%) had atypical chest pain, 9 patients (36%) had typical chest pain, and 11 patients (44%) had shortness of breath. The results of GMPS in 12 patients (48%) were normal, 8 patients (32%) had ischemia, and 5 patients (20%) showed evidence of both ischemia and infarction. The maximum and minimum summed stress scores (SSS) in these 25 patients were 32 and 0 (mean = 7.12), and the maximum and minimum summed rest scores (SRS) and summed difference score (SDS) were 24 and 0 (mean = 3.44 and 3.68), respectively.

The end systolic volume (ESV), end diastolic volume (EDV), and mean filling rate over the first third of diastole (MFR/3) indices obtained by the QGS and QBS software were not correlated. The calculated time to peak filling rate (TTPF), ESV, EDV, and MFR/3 indices showed no significant difference, and there was a significant correlation between the QGS and QBS software ( *p* = 0.523 for TTPF and *p* = 0.00 for others; Table 1 )

**Table 1:** Descriptive output, correlation, and mean difference of LV indices calculated with QGS and QBS

LV indices and methods	Mean	Standard deviation	Correlation ( *p* -value)	Mean difference ± SD ( *p* -value)
ESV and QGS	30.04	24.691	0.882 (0.000)	–15.240 ± 16.310 (000.)
ESV and QBS	45.28	33.206		
EDV and QGS	79.64	28.190	0.827 (0.000)	–36.920 ± 16.437 (000.0)
EDV and QBS	116.56	27.720		
MFR/3 and QGS	1.3504	0.48686	0.599 (0.002)	–0.48760 ± 0.67893 (0.001)
MFR/3 and QBS	1.8380	0.84770		
TTPF and QGS	160.28	33.858	–0.117 (0.576)	9.400 ± 72.532 (0.523)
TTPF and QBS	150.88	60.294		

Abbreviations: EDV, end diastolic volume; ESV, end systolic volume; LV, left ventricle; MFR/3, mean filling rate over the first third of diastole; QBS, quantitative blood pool SPECT; QGS, quantitative gated SPECT; SD, standard deviation; SPECT, single-photon emission computed tomography; TTPF, time to peak filling rate.

Analysis of variance using Wilks' lambda statistics showed significant differences in the calculated LVEF using QGS, QBS, and planar gated blood pool (PGBP; *p* < 0.001). However, in post hoc Greenhouse–Geisser test, LVEF only between QGS and QBS software was not significantly different. The LVPER was also compared between the three software programs (QGS, QBS, and PGBP). The LVPERs of the QBS and QGS methods were similar ( *p* > 0.05), but the LVPERs calculated by the QGS and QBS methods were significantly different from those derived by the PGBP method ( *p* < 0.001).

Based on paired comparisons, the LVPFR variables calculated using QBS and QGS were not significantly different. However, there were significant differences between the LVPFR variable values obtained by QGS and PGBP as well as those obtained by QBS and PGBP. The measured LVEF was significantly correlated ( *p* < 0.05) in the three software. The PER values calculated with QGS and QBS ( *p* < 0.001), as well as PGBP and QGS ( *p* = 0.031), were significantly correlated ( Tables 2 and 3 ).

**Table 2:** Descriptive output of LV indices calculated with QGS, QBS, and PGBP methods

LV indices	Methods	Mean	Standard deviation
EF	QGS	67.800	3.713
	QBS	64.560	3.969
	PGBP	41.712	2.403
PER	QGS	–3.326	0.269
	QBS	–2.960	0.239
	PGBP	583.560	22.509
PFR	QGS	2.645	0.282
	QBS	2.691	0.285
	PGBP	560.880	34.976

Abbreviations: EF, ejection fraction; LV, left ventricle; PER, peak ejection rate; PFR, peak filling rate; PGBP, planar gated blood pool; QBS, quantitative blood pool SPECT; QGS, quantitative gated SPECT.

**Table 3:** Correlation and difference of LV indices calculated with QGS, QBS, and PGBP methods

LV indices and methods			Pearson's correlation ( *p* -value)	Mean differences ± SE ( *p* -value)
EF	QGS	QBS	0.854 (0.000)	3.240 ± 2.093 (0.808)
		PGBP	0.741 (0.000)	26.088 ± 2.518 (0.000)
	QBS	PGBP	0.881 (0.000)	22.848 ± 2.174 (0.000)
PER	QGS	QBS	0.690 (0.000)	–0.367 ± 0.202 (0.082)
		PGBP	–0.431 (0.031)	–586.886 ± 22.626 (0.000)
	QBS	PGBP	–0.369 (0.069)	–586.520 ± 22.598 (0.000)
PFR	QGS	QBS	0.689 (0.000)	–0.046 ± 0.224 (1.000)
		PGBP	0.083 (0.692)	–558.235 ± 34.954 (0.000)
	QBS	PGBP	–0.031 (0.882)	–558.189 ± 34.986 (0.000)

Abbreviations: EF, ejection fraction; LV, left ventricle; PER, peak ejection rate; PFR, peak filling rate; PGBP, planar gated blood pool; QBS, quantitative blood pool SPECT; QGS, quantitative gated SPECT; SE, standard error.

## Discussion

The GMPS method is a valuable tool that can depict both perfusion and myocardial function simultaneously, and assess systolic and diastolic indices with existing software such as QGS.

W showed there was a correlation between QGS, QBS, and PGBP in the calculation of the LVEF. There was no significant difference between QGS and QBS.

There was a correlation but no significant difference between QGS and QBS in the calculation of the LVPFR and LVPER.

Between QGS and QBS, there was significant difference in calculating the EDV, ESV, and MFR/3.

LV time to peak filling (LVTTPF or LVTPF) was not correlated with any method, but there was no significant difference between this parameter and PGBP, QGS, and QBS.

In another study, the LV diastolic factors were compared between the 32-frame gated SPECT and the multigated ERNA. The authors concluded that the LV functional parameters (LVEDV, LVESV, LVEF, and LVPFR) obtained from a 32-frame gated SPECT imaging were closely consistent with the findings of ERNA; hence, they concluded that the diastolic indices derived from a 32-frame gated SPECT are reliable indicators for assessing cardiovascular diseases. 14

Kikkawa et al also compared the LV diastolic parameters obtained using the 32-frame gated SPECT method with findings from ERNA. They concluded there was no significant difference between these parameters obtained using the two methods, and 32-frame gated SPECT can reliably replace ERNA. So early detection of any abnormality in diastolic indices derived from the 32-frame gated SPECT (GSPECT) has great value to prevent systolic dysfunction. They concluded that QGS program with 32 R-R as compared with the 8 or 16 R-R-interval GSPECT was better and suitable for assessment of both LV systolic and LV diastolic functions. 11

Similarly, Acampa et al showed that in all patients (patients with normal or abnormal perfusion scans), there was a good correlation between the LVEF obtained from GSPECT and ERNA in the rest phase; however, in patients with abnormal perfusion scans, the poststress LVEF was underestimated compared with ERNA. 19

We found that only LVEF was correlated between the two ERNA methods (planar and SPECT), and the rest of the indices (PER and PFR) were not correlated. There was also a significant difference between these indices, except for the LVEF. Few studies have examined indices such as LVPER and LVPFR in PGBP scans compared with the gold standard methods such as Cardiac MRI (CMR), and most of the existing studies are limited to the assessment of LVEF in PGBP scans. According to the results of our study, PGBP may be less useful for clinical applications and practice. Further comparative studies are recommended in this regard. 20

The reasons for some of our conflicting results may be due to small sample size, presence of perfusion defects in some patients, and gating by 16-frame gated images. Previous studies compared the 8-, 16-, and 32-frame GSPECT in determining the systolic and diastolic parameters of the LV resulting from GMPS. They observed a higher precision for more frame gating and 16-frame gated images were found to be better than 8-frame gated images. They concluded that variables derived from the 16-frame gated images were suitable for clinical use. 9 12 13

*New Knowledge Gained:* According to our assessment, the LV diastolic functional indices derived from the 16-frame GMPS can be valuable in the serial follow-up and monitoring of cardiovascular diseases.

## Conclusion

By applying 16 frames per cardiac cycle in the resting phase of the cardiac perfusion scan and using available software, the LV functional indices can be measured and applied with sufficient confidence to determine the patient's cardiac function and follow the patients reliably. The functional indices derived from GMPS are significantly correlated with those obtained from ERNA. So in patients who already had a GMPS, physicians can evaluate perfusion and the LV systolic and diastolic functional indices as an alternative to ERNA and not performing further ERNA.
